# Investigating the association between discrimination, internalizing symptoms, and alcohol use among Latino/a immigrants in the United States

**DOI:** 10.3389/fpsyt.2024.1358648

**Published:** 2024-06-05

**Authors:** Miracle A. Macias Burgos, Tara G. Bautista, Yesenia Cruz-Carrillo, Mia Cisco, Sumeyra Sahbaz, Lea Nehme, Duyen Vo, Maria Duque, Seth J. Schwartz, Pablo Montero-Zamora

**Affiliations:** ^1^ Department of Psychological Sciences, Northern Arizona University, Flagstaff, AZ, United States; ^2^ Department of Psychology, Arizona State University, Tempe, AZ, United States; ^3^ University of Texas at Austin, Department of Kinesiology and Health Education, Austin, TX, United States; ^4^ FIU, Department of Epidemiology, Florida International University, Miami, FL, United States

**Keywords:** Latinos, discrimination, alcohol, immigrants, depression, anxiety

## Abstract

Internalizing symptoms associated with anxiety and depression have been correlated with harmful alcohol use among Latino/as, but little attention has been paid to assessing the association between perceived discrimination and harmful alcohol use. The present study was designed to investigate the association between perceived discrimination, internalizing symptoms associated with anxiety and depression, and harmful alcohol use among Latino/a immigrants living in the United States (US). Our sample included 426 Latino/a immigrants. Their mean age was 40.05 years (*SD* = 6.50), 65.50% were women, 80.00% had a partner, 54.20% lived on less than $2,000 a month, and 41.50% reported having attained a college degree. Perceived discrimination was assessed using the Perceived Discrimination Scale, anxious symptoms were assessed using the GAD-7, depressive symptoms were assessed using the CES-D short form, and harmful alcohol use was assessed using AUDIT. We estimated a linear regression model using cross-sectional, self-reported data. The model was statistically significant, *R* ^2^ = 0.38, *F* (8, 425) = 32.09, *p* < 0.01. Discrimination was significantly associated with AUDIT scores, (β = 0.21, *p* < 0.01) after accounting for covariates and for symptoms of anxiety and depression. Our results indicate that experiences of discrimination in the US are associated with increased harmful alcohol use even after controlling for other variables. These findings may be useful in designing coping interventions specifically for Latino/a immigrants to reduce the risk of alcohol use disorder. This study also has political and public health implications for acknowledging the detrimental health consequences of experiencing discrimination, providing support to the position that reducing racism and discrimination represent important public health priorities.

## 1 Introduction

There are 62 million Latino adults, of which 11.1 million report binge drinking in the past month (1, 2). Latinos also have the second highest rate of heavy alcohol use among adult racial and ethnic groups in the U.S. as of 2021, behind non-Hispanic Whites and had the highest age-adjusted death rates attributable to alcohol in the U.S. between 1999–2015 (3, 4). Since 2010, there has been a rise in alcohol use prevalence among Latinos in the U.S. (3) which is particularly concerning because current alcohol misuse treatments strategies are less likely to be utilized by Latinos and may not be appropriately adapted to accommodate their unique cultural values (5). Currently, most available treatments include medications, Alcoholics Anonymous (AA), residential rehabilitation, talk therapy, and cognitive behavioral therapy (6). Due to its accessibility, the most sought treatment, AA, has many limitations including the stigma among Latino immigrant men for seeking treatment to help with their alcohol use (severe and non-severe alcohol use) which in turn reduces the utilization of treatments such as AA (7). Additionally, accessibility to treatment has been cited as another barrier to treatment and among the Latino immigrant men that do seek out treatments like AA there is still perceived stigma associated with seeking out the treatment, possibly contributing to reduced efficacy (5, 7). One explanation for Latino immigrants not seeking alcohol use treatment may also be lack of cultural diversity in treatment to make it more accessible for this group. Therefore, due to stigma and a potential lack of culturally sensitive treatment options, prevention efforts warrant increased attention. Alcohol use may have different antecedents among Latinos than among Whites, but these determinants are not well documented in the literature, including when sociodemographic characteristics are well accounted for (8, 9). It is therefore essential to explore risk factors that may impact alcohol misuse in this population.

Prior research has identified associations between internalizing symptoms such as anxiety and depression and alcohol use among Latino adolescents, young adults, and adults (10–13). Internalizing symptoms such as anxiety symptoms and depression symptoms have been well accounted for in prior literature. Among Latino immigrants there are associations between depression and anxiety symptoms and increased alcohol use which makes depression symptoms and anxiety symptoms important when considering harmful and hazardous alcohol use (14). To the best of our knowledge, although there is extensive research on the relationship between discrimination (i.e. perceived discrimination) and mental health outcomes such as anxiety and depression (15), there remains a significant gap in exploring the specific association of discrimination and harmful and hazardous alcohol use, particularly when accounting for the influence of internalizing symptoms such as anxiety and depression, in the context of adult Latino immigrants residing in the United States. Furthermore, socially based stressors play a significant role in developing vulnerability to substance use disorder (16). For example, discrimination has been identified as an increasing the risk of alcohol use disorder (AUD) and substance use disorder (SUD) among Latino adults (16).

Discrimination (i.e. racial and ethnic discrimination) represents an emerging risk factor for disease and a contributor to health disparities (17). Pascoe and Smith (18) highlighted the role of discrimination (i.e. racial, ethnic and gender discrimination) in producing heightened stress responses and influencing health behaviors, and they emphasized the need for more studies to understand the potential pathways between perceived discrimination and negative health outcomes. Discrimination (i.e. racial discrimination) has been found to lead to internalizing and externalizing symptoms, which in turn are related to substance use and poor physical health outcomes (19). In studies among African Americans, discrimination (i.e. every day, lifetime and perceived discrimination) has been associated with increased engagement in unhealthy behaviors, such as smoking and high-fat diets (20, 21). Similarly, in research among Latino immigrants, Andrade et al. (22) and Cariello et al. (23) both found perceived discrimination to be associated with poorer physical and mental health outcomes, which emphasizes the need for studies to parse out mental health symptoms from alcohol use outcomes. For example, (24) found that anti-Latino discrimination may be linked to poorer sleep quality. This finding is of particular concern considering the increasing prevalence of anti-Latino immigrant sentiment in recent years (25). Furthermore, Latino Americans, when compared with other minoritized groups, are often more affected by perceived discrimination (26). Given this conjecture, discrimination may undermine Latino/a immigrants’ health through the ways in which it interacts with their stress-coping responses and unhealthy behaviors.

Additionally, much prior work has focused on the impact of sociocultural factors (e.g., bicultural stress) on alcohol misuse among immigrant Latino adults and adolescents (27, 28, 29). Oftentimes, perceived discrimination is grouped into the category of sociocultural stressors and examined as a single entity, rarely is perceived discrimination assess as an independent contributing factor to alcohol use among Latino immigrants (30). When individuals consistently experience social stressors in their everyday lives, the resulting overactivated stress cycle is associated with increased prevalence of chronic diseases and vulnerability to substance use (16). For example, engaging in negative coping strategies, such as excessive alcohol consumption, may accelerate rates of disease and disability and represent a public health concern (18, 31). Understanding perceived discrimination, specifically, provides insight on how Latino immigrants’ daily interactions in the U.S. may impact coping mechanisms such as alcohol use (10, 16).

Our study is both supported by the socioecological framework and the cultural stress theory which both consider external factors and internal factors for an individual’s behavior, in this case harmful and hazardous alcohol use. The socioecological framework lets us understand that macro external issues such as discriminatory policy or discrimination can impact a community and further impact an individual’s internal issues such as well-being, health and mental health (16, 32). In that same light, the cultural stress theory explains that migrants may experience cultural stress in three main forms, discrimination, context of reception and bicultural stress which may be associated with that individual’s alcohol misuse (33). As defined by Phinney et al., perceived discrimination in this study will be defined as an individual’s interpretation of events as discriminatory (1998). Further, when individuals are faced with external factors that may impact their mental health and wellbeing, they may look to cope with these feelings which may not be healthy, in this case harmful and hazardous alcohol use. This study using the socioecological framework and the cultural stress theory can inform us if perceived discrimination is associated with increased harmful and hazardous alcohol use when accounting for internalizing symptoms such as anxious and depressive symptoms. In other words, if the external factor of perceived discrimination is associated with the individual’s behavior of harmful and hazardous alcohol use among Latino/a immigrants our goal would be to unpack this relationship (15, 34, 35).

The purpose of the present study was to assess the association between perceived discrimination and harmful and hazardous alcohol use after accounting for anxiety symptoms and depression symptoms among Latino/a immigrants in the United States. We hypothesized that symptoms of anxiety and depression would be strongly associated with harmful and hazardous alcohol use and that, above and beyond these associations, perceived discrimination would account for additional variance in alcohol use among Latino/a immigrants.

## 2 Materials and methods

### 2.1 Participants and procedures

This was a secondary data analysis of the Community Health Research and Implementation Science data (CHeRISh) which was focused on Latino/a immigrant parent health. Four hundred twenty-six (N=426) Latino/a immigrants residing in the U.S. were surveyed using a third-party survey panel company. The survey panel was comprised of participants who have already agreed to partake in survey research. These potential participants were to create an account with the third-party company and were given a list of potential surveys they could complete. Following this participant selected preferred language and filled out a screener questionnaire for eligibility. The survey was online, self-administered and took approximately 15–19 minutes to complete. Our inclusion criteria focused on participants who self-identify as Hispanic or Latino/a, are 18 years or older, are parents and reside in the United States. Our participants were compensated by the web panel which could be different depending on what the participant selected. Demographics variables were collected by self-report including participants’ age, sex, income (less than $200 dollars, 200–499 dollars, 500–999 dollars, 1,000–1,999 dollars, or 2,000 dollars or more), education (no formal education, 1 – 5 grade, 6 – 8 grade, high school, GED equivalent, some college/university, or college/university degree), and marital status (single never married, in a domestic partnership, married, separated, divorced, or widowed). Income was recoded as “less than” $2,000 dollars/month or “more than” $2,000/month. Education was recoded to no college degree or college degree. Marital status was recoded as partner or no partner. Of these 426 participants, the country of origin most represented was Mexico with 49.10% of the sample, the other half was divided into small groups from Ecuador, Venezuela, Colombia, El Salvador and others. The size of groups from each country of origin was too small to make meaningful comparisons. Most participants (80.00%) had a partner (husband, wife, significant other), 65.50% of the participants were female, and 41.50% had a college degree. See **Table 1** for more extensive demographic information. This study was approved by the Institutional Review Board at The University of Texas at Austin. The study was conducted in accordance with local legislation and institutional requirements. Participants provided informed consent to participate in this study.

**Table 1:** Sociodemographic of Participants.

Variable (N = 426)	Mean (SD) or N (%)
Age (years)	40.05 (6.49)
Sex (male)	147 (34.50%)
Education No College Degree College/University Degree	249 (58.50%) 177 (41.50%)
Family Monthly Income Lived on less than 2,000 last month	231 (54.20%)
Marital Status Married/Life Partner Single/Divorced/Separated/Widowed	85 (80.00%) 341 (20.00%)
Depression	18.19 (5.87)
Anxiety	12.31 (5.47)
Discrimination	16.37 (6.64)
Alcohol Use	6.46 (6.91)

### 2.2 Measures

Center for Epidemiologic Studies Depression Boston Form (CES-D; 36, 37). The CES-D Boston form is a 10-item self-reported scale used to assess symptoms of depression. Within the CES-D Boston form there are three factors of depressive symptomology captured; depressed affect and interpersonal relations, somatic activity, and positive affect (37). A sample question of the CES-D short form is “Please indicate how often you felt the following over the last week: I feel depressed” on a scale scored from 0 (rarely or none of the time) to 4 (almost all the time). Our study created a sum score to represent each participant’s symptoms of depression which is the protocol regarding the CES-D Boston form. The CES-D Boston form has been previously validated among Latino/a samples (37). Within this sample, CES-D Boston form scores evidenced strong internal consistency (α= 0.88).

General Anxiety Disorder Scale (GAD-7; 38). The 7-item GAD-7 was used to measure anxiety symptoms. Each item in the GAD-7 was reported to measure the concept of GAD equally, therefore item 1 measured nervousness, item 2 measured stop worrying, item 3 measured excessively worried, item 4 measured restlessness, item 5 measured difficulty in relaxing, item 6 measured irritableness, and item 7 measured being afraid of something awful (39). A sample question from the GAD-7 is “In the last 2 weeks, how often have you been bothered by the following: feeling nervous, anxious or on edge” and is scored from 0 (not at all) to 4 (nearly every day). Additionally, the GAD-7 which measured anxiety symptoms in this study was made into a sum score to represent the participants’ symptoms of anxiety. GAD-7 scores evidenced strong reliability (α= 0.93).

Perceived Discrimination Scale (PDS; 40). The Perceived Discrimination Scale was used to assess negative and unfair experiences in the U.S. perceived to be related to their identity as a minoritized group. The Perceived Discrimination scale has three items measuring perceived frequency of being treated unfairly or negatively because of their ethnic background and four items measuring feeling unaccepted in society because of their ethnic identity (40) The measure uses a Likert scale ranging from 1 (never) to 5 (almost always). A sample question is “How often do other people (such as police and shopkeepers) treat you unfairly or negatively because you are Latino?”. We added each item to create a sum score to represent the participants’ discrimination they reported. The perceived discrimination scale showed strong internal consistency (α= 0.93).

Alcohol Use Disorders Identification Test (AUDIT; 41). The AUDIT is a scale utilized to screen for hazardous and harmful alcohol consumption. In the AUDIT, questions 1–3 assess alcohol consumption, questions 4–6 assess drinking behavior, questions 7–8 assess adverse reactions and questions 9–10 assess alcohol-related problems (41). It uses a response scale ranging from 0 (never/no) to 4 (4 or more times a week/yes during the past year) for questions 1 through 8; and response options for question 9 and 10 were 0 (No), 2 (Yes, but not in the past year), or 4 (Yes, during the past year). In our analysis we created a sum score to represent each participant’s AUDIT score for harmful and hazardous alcohol use. A sample AUDIT question includes “have you or someone else been injured as a result of your drinking?” The AUDIT provided scores with strong internal consistency (α= 0.88).

### 2.3 Data analytic plan

SPSS version 28 (IBM Corp., Armonk, NY) was used for all data cleaning and analyses. Bivariate Pearson correlations, t-tests, and analysis of variance (ANOVA) were computed to examine associations between independent variables, demographic variables and the outcome variable to identify the potential inclusion of necessary covariates. The demographic variables that were significantly associated with the independent variables, such as age, sex, and income, were included as covariates in the models. In addition to these demographic variables, we also included education and marital status (whether or not the participant had a partner) as covariates based on the theoretical support that these variables may be associated with our independent variables and on similar research studies that include these variables as covariates (42, 43). Sum scores were computed for the AUDIT, perceived discrimination scale, CES-D, and GAD-7.

Upon examination of dependent variable distributions and model metrics, multiple linear regression was selected to appropriately fit the data. Model assumptions for linear regression were tested by assessing model residuals, Cook’s distance indicates whether a single case influences our model as a whole and in this case an influential case would exceed a test statistic of 1 for Cook’s distance. The maximum calculated for our data was 0.05, indicating that there was no single influential case on our model as a whole. Our maximum statistic for our regression analysis using leverage was 0.06. The data presented no issues or violations involving any assumptions of normality necessary for regression analyses. A multiple linear regression model was estimated to examine the associations of depressive symptoms, anxiety symptoms, and perceived discrimination with harmful and hazardous alcohol consumption among Latino/a immigrants. Despite the independent variables being significantly correlated with each other, there were no issues with multicollinearity in the regression model; all variance inflation factor values (VIF) were below 3.00 (CES-D tolerance = 0.34, VIF = 2.91; GAD-7 tolerance = 0.37, VIF = 2.67; Discrimination tolerance = 0.75, VIF = 1.33).

## 3 Results

### 3.1 Bivariate relationships

AUDIT scores differed significantly between males (M = 9.53, SD = 8.24) and females (M = 4.83, SD = 5.44); *t* (214.83) = 6.23, two-tailed *p* <.01). There was also a significant difference in AUDIT scores for participants making less than $2,000/month (M = 7.61, SD = 7.51) and participants making $2,000/month or more (M = 5.09, SD = 5.85); *t* (421.36) = 3.89, two-tailed *p* <.01). No significant difference emerged for AUDIT scores between participants with a partner (married/living with a significant other) versus without a partner (single/divorced/widowed). No significant difference was present for AUDIT scores between participants with a college degree (college/university degree) versus no college degree (no formal education, 1 – 5 grade, 6 – 8 grade, high school, GED equivalent, some college/university). Participant age was negatively associated with AUDIT scores were (*r* = -0.10, *p* <.05). CES-D scores were moderately and positively associated with AUDIT scores (*r* = .46, *p* <.01), and GAD-7 scores were moderately and positively associated with AUDIT scores (*r* = .45, *p* <.01). Lastly, perceived discrimination scores were moderately and positively associated with AUDIT scores (*r* = .41, *p* <.01). See **Table 2** for all bivariate correlation coefficients.

**Table 2:** Bivariate Pearson Correlation.

Variable	CES-D	GAD-7	Discrimination	AUDIT
*1. Age*	-0.18**	-0.13**	-0.14**	-0.10*
*3. CES-D*	–	0.79**	0.47**	0.46**
*4. GAD-7*		–	0.40**	0.45**
*5. Discrimination*			–	0.41**
*6. AUDIT*				–

**p <0.01, *p < 0.05.

### 3.2 Primary analyses – linear regression

Based on bivariate results with the current sample and covariate inclusion in prior similar studies, we included age, education, marital status, sex, and income as covariates for the model with CES-D, GAD-7, and perceived discrimination scores as independent variables and AUDIT scores as the dependent variable. The overall model was significant (*R* ^2^ = .38, *F* (8, 425) = 32.09, *p* <.01). A small positive association emerged between GAD-7 and AUDIT (β = .21, *p* <.01). CES-D scores were also small and positively associated with AUDIT scores (β = .19, *p* < 0.05). Finally, discrimination scores were small and positively association with AUDIT scores (β =.20, *p* <.01). Please see Table 3 for the overall linear regression model (**Table 3**).

**Table 3:** A regression model with hazardous and harmful alcohol consumption (AUDIT) as the dependent variable.

Overall Model: *R^2^ =*0. 38**		*β*	*SE β*
Covariates	Age	-0.01	0.04
	Education	0.01	0.56
	Marital Status	0.05	0.69
	Sex (Male)	-0.30	0.58
	Income	-0.07	0.56
Depression		0.19**	0.07
Anxiety		0.21**	0.08
Discrimination		0.20**	0.05

**p < 0.01.

β = linear regression standardized effect size estimate.

SE β = standard error of standardized effect size.

The linear regression model with harmful and hazardous alcohol use as the dependent variable has three independent variables after controlling for covariates.

## 4 Discussion

Although previous work has documented an association between psychosocial stressors and alcohol use, the contribution of discrimination-specific stress, above and beyond symptoms of anxiety and depression, has not been widely explored. The present study provides a novel perspective on the distinct association between perceived discrimination and harmful and hazardous alcohol consumption among Latino/a immigrant adults living in the United States. Our study aimed to bridge this gap in knowledge by examining the association between perceived discrimination and harmful and hazardous alcohol use beyond what is explained by symptoms of anxiety and depression among Latino/a immigrants. As hypothesized, perceived discrimination was significantly and positively associated with harmful and hazardous alcohol use among Latino/a immigrants, even when accounting for depressive and anxious symptoms.

Our results are consistent with other studies examining the association between cultural stressors and harmful and hazardous alcohol use. For example, (44) reported that greater levels of cultural stress were associated with increased severity of alcohol use among Latino immigrant men in Miami-Dade County, Florida. Our study adds to the growing evidence that perceived discrimination at the individual level is associated with poor mental health outcomes (34, 35, 45). As a result, our findings at the individual level can be used to inform public health interventions which are directed toward Latino/a immigrant health, especially in terms of integrating individual health findings to community level programs.

Results from the present study should be interpreted considering certain limitations. First, our cross-sectional design does not permit us to draw any causal inferences. Second, while our study was not focused on parenting, our data was pulled from an existing sample consisting of Latino/a immigrant parents with a mean age of 40 – and as a result, our results may not be generalizable to non-Latino/a immigrants, younger Latino/as, or immigrants without children as these various groups may have different characteristics such as age at migration, acculturation, and language abilities and barriers (46–48). Future studies may also consider assessing parenting stress and social support as potential variables of interests in explaining harmful and hazardous alcohol use among Latino/a immigrant parents in the United States. Due to our sample size and sample distribution, we could not disaggregate Latino/a immigrants from their country of origin to explore potential differences in harmful and hazardous alcohol use between Latino/a countries. This limitation is particularly important because harmful and hazardous alcohol use such as binge drinking has been reported to differ by sex but also Latin American country of origin, specifically that Mexican and Puerto Rican Latino/as have higher binge drinking patterns compared to South Americans (49).

Despite these limitations, our findings suggest a need for intervention efforts in Latino/a health to include recognizing discrimination or potentially discriminatory policies to offset the effect of discrimination on harmful and hazardous alcohol use. Addressing discrimination against Latinos/a immigrants may improve mental health outcomes and, as our results suggest, may reduce harmful and hazardous alcohol consumption. One qualitative study found that among older gay men, when faced with discrimination over the years, built resilience through sustained social networks, addressing their mental health and advocating for themselves (50). Future studies may examine strategies to prevent discrimination towards Latinos/a immigrants of all ages. Our findings among Latino/a immigrant adults complements other evidence that has shown that everyday discrimination negatively influences developmental health outcomes in children (45). Mental health outcomes such as depression and anxiety are also associated to immigration-related discrimination in a sample of Latino/a undergraduate college students, regardless of if the discrimination was directed toward the student’s immigration status or the parents (51) Efforts should be undertaken to change discriminatory policies like SB1070 in Arizona which allows law enforcement to enact anti-immigration laws to assist immigration officers (35). To help counteract policies that weaken well-being or increase internalizing symptoms such as anxiety and depression symptoms among Latino/a immigrants’ future goals would be to improve access to interventions designed to improve health outcomes among Latino/a immigrants.

In conclusion, the purpose of the present study was to examine perceived discrimination, specifically among Latino/a immigrants, as a potential risk factor for harmful and hazardous alcohol use after accounting for symptoms of depression and anxiety. Our results highlight the importance of assessing perceived discrimination within studies targeting harmful and hazardous alcohol use. Future work should explore the complex interplay between perceived discrimination, mental health, and harmful and hazardous alcohol use, which could lead to more effective interventions and support for vulnerable populations.

## Data availability statement

The raw data supporting the conclusions of this article will be made available by the authors, without undue reservation.

## Ethics statement

The studies involving humans were approved by Institutional review board: The University of Texas at Austin. The studies were conducted in accordance with the local legislation and institutional requirements. The participants provided their written informed consent to participate in this study. Written informed consent was obtained from the individual(s) for the publication of any potentially identifiable images or data included in this article.

## Author contributions

MM: Writing – review & editing, Writing – original draft, Methodology, Investigation, Formal analysis, Conceptualization. TB: Writing – review & editing, Writing – original draft, Visualization, Supervision, Methodology, Investigation, Formal analysis, Conceptualization. YC: Writing – review & editing, Writing – original draft, Conceptualization. MC: Writing – review & editing, Writing – original draft. SS: Writing – original draft, Writing – review & editing. LN: Writing – original draft, Writing – review & editing. DV: Writing – review & editing, Writing – original draft. MD: Writing – review & editing. SS: Supervision, Writing – review & editing. PM: Visualization, Supervision, Resources, Project administration, Methodology, Investigation, Funding acquisition, Formal analysis, Data curation, Conceptualization, Writing – review & editing, Writing – original draft.

## References

[1] U.S. Census Bureau. Race and Ethnicity in the United State; 2010 Census and 2020 Census . Available at: https://data.census.gov/.

[2] SAMHSA. Center for Behavioral Health Statistics and Quality. 2022 National Survey on Drug Use and Health. Table 2.28A—Binge alcohol use in past month: among people aged 12 or older; by age group and demographic characteristics, numbers in thousands, 2021 and 2022 . Available at: https://www.samhsa.gov/data/sites/default/files/reports/rpt42728/NSDUHDetailedTabs2022/NSDUHDetailedTabs2022/NSDUHDetTabsSect2pe2022.htm#tab2.28a.

[3] SAMHSA Center for Behavioral Health Statistics and Quality. National Survey on Drug Use and Health. Table 2.28B—Binge alcohol use in past month: among people aged 12 or older; by age group and demographic characteristics, percentages, 2021 and 2022 (2022). Available at: https://www.samhsa.gov/data/sites/default/files/reports/rpt42728/NSDUHDetailedTabs2022/NSDUHDetailedTabs2022/NSDUHDetTabsSect2pe2022.htm#tab2.28.

[4] QuickStats. Age-adjusted death rates attributable to alcohol-induced causes, by race/ethnicity — United States 1999–2015. MMWR. Morbidity Mortality Weekly Rep. (2017) 66:491–1. doi: 10.15585/mmwr.mm6618a12 PMC5657989 28493858

[5] Pinedo M Zemore S Rogers S. Understanding barriers to specialty substance abuse treatment among Latinos. J Subst Abuse Treat. (2018) 94:1–8. doi: 10.1016/j.jsat.2018.08.004 30243409 PMC6157272

[6] NIAAA. What types of alcohol treatment are available? National Institute on Alcohol Abuse and Alcoholism (2023). Available at: https://alcoholtreatment.niaaa.nih.gov/what-to-know/types-of-alcohol-treatment.

[7] Carey CM. Help-seeking patterns and perceived barriers to care among latino immigrant men with unhealthy alcohol use. University of Washington: ProQuest Dissertations Publishing (2019).

[8] Haeny AM Oluwoye O Cruz R Iheanacho T Jackson AB Fisher S. Drug and alcohol treatment utilization and barriers among black, American Indian/alaskan native, Latine, Asian/pacific islander/native Hawaiian, and white adults: Findings from NESARC-III. J Subst Abuse Treat. (2021) 131:108569. doi: 10.1016/j.jsat.2021.108569 34393011 PMC9084614

[9] Pinedo M Castro Y Gilbert PA Caetano R Zemore SE. Improving assessment of alcohol treatment barriers among Latino and white adults with an alcohol use disorder: Development of the barriers to specialty alcohol treatment scale. Drug Alcohol Depend. (2023) 248:109895. doi: 10.1016/j.drugalcdep.2023.109895 37156194 PMC10802933

[10] Torres L Ong AD. A daily diary investigation of Latino ethnic identity, discrimination, and depression. Cultural Diversity Ethnic Minority Psychol. (2010) 16:561–8. doi: 10.1037/a0020652 21058819

[11] Schwartz Unger JB Baezconde-Garbanati L Zamboanga BL Lorenzo-Blanco EI Des Rosiers SE. Trajectories of cultural stressors and effects on mental health and substance use among Hispanic immigrant adolescents. J Adolesc Health. (2015) 56:433–9. doi: 10.1016/j.jadohealth.2014.12.011 PMC4399639 25650112

[12] Cabrera Tineo YA Dillon FR Ertl MM Rentería R De La Rosa M. Discrimination-based acculturative stress, depression, and alcohol use among Latina emerging adults during initial months in the USA. Int J Ment Health Addict. (2022) 20:553–68. doi: 10.1007/s11469-020-00386-x PMC8937027 35321450

[13] Schwartz S Montero-Zamora P Salas-Wright CP Brown EC Garcia MF Scaramutti C. After hurricane Maria: effects of disaster trauma on Puerto Rican survivors on the US mainland. psychol Trauma. (2022). doi: 10.1037/tra0001371 PMC11077626 36174152

[14] Platt R Arribas-Ibar E. 2329: Psychosocial risk factors mental health symptoms, and intervention preferences of Latino immigrant parents presenting to a pediatric clinic. J Clin Trans Sci. (2018) 1:73–4. doi: 10.1017/cts.2017.260

[15] Andrade N Ford AD Alvarez C. Discrimination and Latino health: A systematic review of risk and resilience. Hispanic Health Care Int. (2021) 19:5–16. doi: 10.1177/1540415320921489 32380912

[16] Amaro H Sanchez M Bautista T Cox R. Social vulnerabilities for substance use: Stressors, socially toxic environments, and discrimination and racism. Neuropharmacology. (2021) 188:108518–8. doi: 10.1016/j.neuropharm.2021.108518 PMC8126433 33716076

[17] Williams DR Lawrence JA Davis BA Vu C. Understanding how discrimination can affect health. Health Serv Res. (2019) 54:1374–88. doi: 10.1111/1475-6773.13222 PMC6864381 31663121

[18] Pascoe EA Smart Richman L. Perceived discrimination and health: A meta-analytic review. psychol Bull. (2009) 135:531–54. doi: 10.1037/a0016059 PMC2747726 19586161

[19] Gibbons FX Kingsbury JH Weng C-Y Gerrard M Cutrona C Wills TA. Effects of perceived racial discrimination on health status and health behavior: A differential mediation hypothesis. Health Psychol. (2014) 33:11–9. doi: 10.1037/a0033857 PMC3893709 24417690

[20] Sims M Diez-Roux AV Gebreab SY Brenner A Dubbert P Wyatt S. Perceived discrimination is associated with health behaviors among African-Americans in the Jackson Heart Study. J Epidemiol Community Health. (2015) 70:187–94. doi: 10.1136/jech-2015-206390 PMC5014355 26417003

[21] Smart Richman L Blodorn A Major B. An identity-based motivational model of the effects of perceived discrimination on health-related behaviors. Group Processes Intergroup Relations. (2016) 19:415–25. doi: 10.1177/1368430216634192

[22] Andrade N Ford AD Alvarez C. Discrimination and Latino health: A systematic review of risk and resilience. Hispanic Health Care Int. (2020) 19:154041532092148. doi: 10.1177/1540415320921489 32380912

[23] Cariello AN Perrin PB Williams CD Espinoza GA Paredes AM Moreno OA. Moderating influence of social support on the relations between discrimination and health via depression in Latinx immigrants. J Latinx Psychol. (2022) 10(2):98–111. doi: 10.1037/lat0000200 PMC9012262 35434535

[24] Slopen N Williams DR. Discrimination, Other Psychosocial Stressors, and Self-Reported Sleep Duration and Difficulties, Sleep, Vol. 37 1. (2014). pp. 147–56. doi: 10.5665/sleep.3326. PMC3865350 24381373

[25] Newman B Shah S Collingwood L. During the election, Donald Trump's racist rhetoric activated the fears of people in areas with growing Latino populations. USApp - Am Politics Policy Blog (2018). http://eprints.lse.ac.uk/id/eprint/88521.

[26] Paradies Y Ben J Denson N Elias A Priest N Pieterse A. ). Racism as a Determinant of Health: A Systematic Review and Meta-Analysis. PloS One 10(9):e0138511–e0138511. doi: 10.1371/journal.pone.0138511 PMC4580597 26398658

[27] Balagopal G Davidson S Gill S Barengo N de la Rosa M Sanchez M. The impact of cultural stress and gender norms on alcohol use severity among Latino immigrant men. Ethnicity Health. (2022) 27:1271–89. doi: 10.1080/13557858.2021.1880550 PMC8363673 33586536

[28] Goldbach JT Berger Cardoso J Cervantes RC Duan L. The relation between stress and alcohol use among Hispanic adolescents. Psychol Addictive Behav. (2015) 29:960–8. doi: 10.1037/adb0000133 PMC4701595 26551265

[29] Meca A Zamboanga BL Lui PP Schwartz SJ Lorenzo-Blanco EI Gonzales-Backen MA. Alcohol initiation among recently immigrated Hispanic adolescents: Roles of acculturation and sociocultural stress. Am J Orthopsychiatry. (2019) 89:569–78. doi: 10.1037/ort0000352 PMC6669121 30702329

[30] Sudhinaraset M Wigglesworth C Takeuchi DT. Social and cultural contexts of alcohol use: influences in a social-ecological framework. Alcohol Research: Curr Rev. (2016) 38:35–45. 10.35946/arcr.v38.1.05 PMC4872611 27159810

[31] Williams DR Mohammed SA. Discrimination and racial disparities in health: evidence and needed research. J Behav Med. (2009) 32:20–47. doi: 10.1007/s10865-008-9185-0 19030981 PMC2821669

[32] Payan T. Understanding the nexus between undocumented immigration and mental health. Curr Opin Psychol. (2022) 47:101414–4. doi: 10.1016/j.copsyc.2022.101414 35926398

[33] Salas-Wright CP Schwartz SJ. The study and prevention of alcohol and other drug misuse among migrants: toward a transnational theory of cultural stress. Int J Ment Health Addict. (2019) 17:346–69. doi: 10.1007/s11469-018-0023-5

[34] Ayón C Becerra D. Latino immigrant families under siege: The impact of SB1070, discrimination, and economic crisis. Adv Soc Work Special Issue: Latinos/Latinas U.S. (2013) 14:206–28. doi: 10.18060/2692

[35] Ayón C García SJ. Latino immigrant parents’ Experiences with discrimination: implications for parenting in a hostile immigration policy context. J Family Issues. (2019) 40:805–31. doi: 10.1177/0192513X19827988

[36] Kohout FJ Berkman LF Evans DA Cornoni-Huntley J. Two shorter forms of the CES-D depression symptoms index. J Aging Health. (1993) 5:179–93. doi: 10.1177/089826439300500202 10125443

[37] Grzywacz JG Hovey JD Seligman LD Arcury TA Quandt SA. Evaluating short-form versions of the CES-D for measuring depressive symptoms among immigrants from Mexico. Hispanic J Behav Sci. (2006) 28:404–24. doi: 10.1177/0739986306290645 PMC6876313 31768090

[38] Spitzer RL Kroenke K Williams JBW Löwe B. A brief measure for assessing generalized anxiety disorder: the GAD-7. Arch Internal Med. (2006) 166:1092–7. doi: 10.1001/archinte.166.10.1092 16717171

[39] García-Campayo J Zamorano E Ruiz MA Pardo A Pérez-Páramo M López-Gómez V. Cultural adaptation into Spanish of the generalized anxiety disorder-7 (GAD-7) scale as a screening tool. Health Qual Life Outcomes. (20102010) 8:8. doi: 10.1186/1477-7525-8-8 20089179 PMC2831043

[40] Phinney JS Madden T Santos LJ. Psychological variables as predictors of perceived ethnic discrimination among minority and immigrant adolescents 1. J Appl Soc Psychol. (1998) 28:937–53. doi: 10.1111/j.1559-1816.1998.tb01661.x

[41] Saunders JB Aasland OG Babor TF de la Fuente JR Grant M. Development of the alcohol use disorders identification test (AUDIT): WHO collaborative project on early detection of persons with harmful alcohol consumption–II. Addict (Abingdon England). (1993) 88:791–804. doi: 10.1111/j.1360-0443.1993.tb02093.x 8329970

[42] Molina KM Estrella ML Durazo-Arvizu R Malcarne VL Llabre MM Isasi CR. Perceived discrimination and physical health-related quality of life: The Hispanic Community Health Study/Study of Latinos (HCHS/SOL) Sociocultural Ancillary Study. Soc Sci Med (1982). (2019) 222:91–100. doi: 10.1016/j.socscimed.2018.12.038 PMC6377306 30623798

[43] Paulus DJ Rodriguez-Cano R Garza M Ochoa-Perez M Lemaire C Bakhshaie J. Acculturative stress and alcohol use among Latinx recruited from a primary care clinic: moderations by emotion dysregulation. Am J Orthopsychiatry. (2019) 89:589–99. doi: 10.1037/ort0000378 30702327

[44] Cano MA Sánchez M Trepka MJ Dillon FR Sheehan DM Rojas P. Immigration stress and alcohol use severity among recently immigrated hispanic adults: examining moderating effects of gender, immigration status, and social support. J Clin Psychol. (2017) 73:294–307. doi: 10.1002/jclp.22330 27228112 PMC5159315

[45] Metzner F Adedeji A Wichmann ML-Y Zaheer Z Schneider L Schlachzig L. Experiences of discrimination and everyday racism among children and adolescents with an immigrant background - results of a systematic literature review on the impact of discrimination on the developmental outcomes of minors worldwide. Front Psychol. (2022) 13:805941. doi: 10.3389/fpsyg.2022.805941 35615177 PMC9126147

[46] Torres Stone RA Meyler D. Identifying potential risk and protective factors among non-metropolitan Latino youth: cultural implications for substance use research. J Immigrant Minority Health. (2007) 9:95–107. doi: 10.1007/s10903-006-9019-5 17136612

[47] Anania A Nageswaran S Miller-Fitzwater A Golden S. Beyond language barriers: caring for Latino children with complex chronic conditions (TH309-C). J Pain Symptom Manage. (2014) 47:392–3. doi: 10.1016/j.jpainsymman.2013.12.037

[48] Torres JM Ro A Sudhinaraset M. Reconsidering the Relationship between Age at Migration and Health Behaviors among US Immigrants: The Modifying Role of Continued Cross-border Ties. Immigration Health. (2019) 19:17–45. doi: 10.1108/S1057-629020190000019002 PMC6330705 30655659

[49] Ramisetty-Mikler S Caetano R Rodriguez LA. The Hispanic Americans Baseline Alcohol Survey (HABLAS): alcohol consumption and sociodemographic predictors across Hispanic national groups. J Subst Use. (2010) 15:402–16. doi: 10.3109/14659891003706357 PMC3705778 23847447

[50] Handlovsky I Bungay V Oliffe J Johnson J. Developing resilience: gay men’s response to systemic discrimination. Am J Men’s Health. (2018) 12:1473–85. doi: 10.1177/1557988318768607 PMC6142121 29683025

[51] Rodriguez VE Enriquez LE Ro A Ayón C. Immigration-related discrimination and mental health among Latino undocumented students and U.S. Citizen students with undocumented parents: A mixed-methods investigation. J Health Soc Behav. (2023) 64:593–609. doi: 10.1177/00221465231168912 37222500 PMC10683331

